# Effect of Aging on Tensile and Chemical Properties of Polylactic Acid and Polylactic Acid-Like Polymer Materials for Additive Manufacturing

**DOI:** 10.3390/polym16081035

**Published:** 2024-04-10

**Authors:** Zorana Golubović, Božica Bojović, Snežana Kirin, Aleksa Milovanović, Ljubiša Petrov, Boban Anđelković, Ivana Sofrenić

**Affiliations:** 1 Faculty of Mechanical Engineering, University of Belgrade, 11120 Belgrade, Serbia; 2 Innovation Center of Faculty of Mechanical Engineering, 11120 Belgrade, Serbia; 3 Faculty of Chemistry, University of Belgrade, 11158 Belgrade, Serbia

**Keywords:** additive manufacturing, 3D printing, PLA, PLA-like resin, fused deposition modeling (FDM), digital light processing (DLP), tensile testing, Fourier transform infrared spectrometry (FTIR), 3D scanning, statistical analysis

## Abstract

Additive manufacturing, with its fast development and application of polymeric materials, led to the wide utilization of polylactic acid (PLA) materials. As a biodegradable and biocompatible aliphatic polyester, produced from renewable sources, PLA is widely used in different sectors, from industry to medicine and science. The aim of this research is to determine the differences between two forms of the PLA material, i.e., fused deposition modeling (FDM) printed filament and digital light processing (DLP) printed resin, followed by aging due to environmental and hygiene maintenance conditions for a period of two months. Specimens underwent 3D scanning, tensile testing, and Fourier transform infrared (FTIR) spectrometry to obtain insights into the material changes that occurred. Two-way Analysis of Variance (ANOVA) statistical analysis was subsequently carried out to determine the statistical significance of the determined changes. Significant impairment can be observed in the dimensional accuracies between both materials, whether they are non-aged or aged. The mechanical properties fluctuated for aged FDM specimens: 15% for ultimate tensile stress, 15% for elongation at yield, and 12% for elastic modulus. Regarding the DLP aged specimens, the UTS decreased by 61%, elongation at yield by around 61%, and elastic modulus by 62%. According to the FTIR spectral analysis, the PLA materials degraded, especially in the case of resin specimens. Aging also showed a significant influence on the elastic modulus, ultimate tensile stress, elongation at yield, elongation at break, and toughness of both materials, which was statistically shown by means of a two-way ANOVA test. The data collected in this research give a better understanding of the underlying aging mechanism of PLA materials.

## 1. Introduction

The fast evolution of additive manufacturing (AM) in recent decades is evident in various areas of industry and research, especially in the growing interest in its implementation in medicine [1,2]. AM enables the production of a broad range of functional components, or prototypes, with complex geometries which can easily be customized, based on specific demands. Among the many types of materials that are used in AM, polymers play a significant role in producing reliable parts with required properties at a low cost. According to their composition, polymer materials are divided into natural, synthetic, and hybrid materials [3]. The range of thermoplastic and thermoset materials is wide, comprising polylactic acid (PLA), Polyamide (PA) and Acrylonitrile Butadiene Styrene (ABS), Polypropylene (PP), Polycarbonate (PC), Polyethylene (PE), Nylon or Polyamide (PA), Polymethyl Methylacrylate (PMMA), and polyether-ether-ketone (PEEK) [4,5]. However, PLA is among the most popular and extensively researched polymer materials so far [6]. According to its chemical composition, PLA is an aliphatic polyester derived from bio-renewable sources (sugar beets, cornstarch, etc.), known for its biocompatibility and biodegradability. After being used, PLA components can easily be recycled through hydrolysis without additional enzymes [7]. The material has significant potential for meeting the requirements in various industrial and medical applications, eventually replacing petroleum-based polymers [8,9,10,11]. PLA boasts great advantages as a structural material, but unfortunately, it has some disadvantages, which are highlighted in many research papers [12].

From the vast number of studies, it is evident that PLA is a thoroughly investigated material for fused deposition modeling (FDM), which is an extrusion-based AM technology that is widely used [13,14]. In FDM, the final parts are manufactured from filament materials which are melted and deposited onto a build platform. before the AM process occurs in the filament form. The main benefits of the FDM process are its simple utilization and the low cost of devices, materials, and any additional equipment [15]. A large number of parameters affect the quality and mechanical/chemical properties of AM parts, and hence, insight into the exact influence of these parameters is crucial for achieving the required part properties [16,17]. Also, PLA parts produced with FDM are influenced by certain factors, e.g., 3D printing devices, filament properties, design, process parameters, post-processing, and aging, as well as the mechanical testing methodology [18].

PLA parts manufactured with this AM process, compared to other thermoplastics, have relatively high tensile strength, elastic modulus, and dimensional accuracy [11,19]. The main drawbacks are inferior ductility resulting in low impact strength and low crystallization rate [20,21]. Studies have shown that FDM-printed PLA is a brittle material, when examined immediately [6] or aged [22,23], and regardless of the usage of water.

PLA can also be in a resin form, and depending on the applications, there are different kinds of materials with varied properties [11]. Data on PLA resins are limited, leaving space for examinations and experiments to comprehensively characterize mechanical and other properties. The constitution of resins can be tailored by controlling the different chemical and molecular parameters during the production process [8,24]. Bearing in mind that resins are mainly used in vat photopolymerization technologies to improve their properties, different changes can be applied via reinforcement agents, blending, copolymerization, fillers, and composite production [25]. However, the literature dealing with PLA-like resin materials for VAT polymerization is limited.

The FDA and European regulatory bodies have approved PLA resins and acknowledged them as a biodegradable polymer material for use in all food packaging, as well as certain surgical applications [26,27,28]. Because of its benefits, PLA is a very adaptable polymer that may be used to create a variety of resins based on the relevant needs [29]. A low impact strength and low fracture toughness are two of PLA resins’ drawbacks, which affect their use in specific applications. Fillers are therefore employed to improve this material’s mechanical properties [30].

Aging is an important factor to keep in mind and influences the engineering properties (i.e., strength, toughness, etc.), physical properties (density, hardness, etc.), or chemical characteristics of polymers over time [31]. Aging can be affected by different environmental conditions, oxidation processes taking place, and chemical processes during curing and cooling, which can appear simultaneously.

AM-manufactured parts can encounter different environmental conditions and loads during the time of use. Any kind of aging, i.e., photo-oxidation during light exposure [32], thermal [33], aging during hydrolysis [34], or natural weathering [35] can lead to structural changes in the AM components [36]. Various studies explored the aging behavior of PLA materials under different conditions, resulting in changes in the mechanical parameters of the examined specimens. Most changes happen because of chemical depolymerization and the degradation of polymers due to exposure to temperature, UV light, water, or chemicals [37,38].

There are many studies dealing with the effect of temperature and relative humidity on the degradation of PLA. The methodology of testing is given in standardized methods for all plastic materials, but the specificities of each material should be taken into account. Hasan et al. realized that thermal aging affects the degradation of PLA specimens and that it is predominant in lower printing resolutions, i.e., in thicker layers [12]. One other study concluded that when subjected to temperature changes, PLA degrades together with its copolymers, where PLA’s thermal stability descends when heated above the melting temperature (Tm). The authors showed that keeping the PLA at 10 °C above its Tm (~160 °C) for a certain period leads to notable molecular degradation [39]. PLA has shown sensitivity to water when it enters the hydrolysis process, leading to a decrease in molecular weight and consequently a degradation of mechanical properties [40,41]. The hydrolysis process occurs both when parts are placed in a high-relative-humidity ambient environment, and when they are immersed in water [42]. Bergaliyeva et al. proposed thermal and hydrothermal aging of PLA specimens produced by means of the FDM process under real conditions of indoor operations. It was found that the shrinkage ratio for hydrothermally aged specimens is greater, while the calorimetry test showed that Tg and Tm are similar for both types of aging [6]. UV radiation, i.e., light exposure, causes photochemical reactions that further influence the degradation of a polymer matrix by cross-linking or chain scission [43,44]. Amza et al. analyzed the effect of accelerated aging through UV-B and UV-C exposure on the mechanical properties of FDM-printed parts made from PLA and PTG materials. It was found that the ultimate tensile strength decreased slightly for PLA specimens undergoing a 24 h UV-B exposure, while in the case of UV-C exposure, the reduction in mechanical properties (ultimate tensile strength, compressive strength) was moderate in PLA parts (6–8%) [45,46]. The aging of PLA specimens was tested by Valerga et al. through exposure to fertilized soil for a period of 6 months. They chemically treated some of the specimens to improve the surface quality, and the effect of aging on the treated and untreated specimens was examined. It was found that the ultimate tensile strength decreased during the time passage from 46 to 36 MPa (22%), and it increased with the treatment time by high percentage values (40%) [47].

Regardless of the multitude of studies that have been carried out to determine the impact of different PLA materials’ aging on various parameters, there are still unexplored areas for research. To the knowledge of the authors of this manuscript, there are no research articles examining the aging of PLA resin materials, nor comparing commercial PLA filaments and commercial PLA-like resins. This study aims to give an insight into the mechanical properties and differences specifically between PLA specimens that are 3D-printed by means of the FDM and DLP processes, followed by aging due to environmental and hygiene maintenance conditions for a period of two months. Specimens underwent 3D scanning, tensile testing, and FTIR spectrometry to gain insights into changes that occurred during the two months. In the end, ANOVA statistical analysis was performed to point out the statistical significance of the determined changes.

## 2. Materials and Methods

### 2.1. Materials and Specimen Preparation

Two commercially available materials were used in this research: PLA filament (Creality, Shenzhen, China) and PLA-like resin (eSUN, Shenzhen, China). Specimen geometry was modeled in CAD software (SolidWorks 2020, Dassault Systèmes SE, Vélizy-Villacoublay, France) according to the specified standard for tensile testing, i.e., ISO 527-2 standard [48]. The model of the specimen was then converted to an STL file format and sliced in the corresponding software for FDM (Simplify3D, Cincinnati, OH, USA) and DLP (ChiTuBox, Shenzhen, China) printers.

In total, there are 30 specimens, i.e., 15 for PLA filament and 15 for PLA-like resin, all with a 90° print orientation, grid infill pattern, and 100% infill density. Specimens were manufactured on the “Creality CR-10 smart pro FDM” for PLA filaments and “Creality LD -002R DLP” for PLA resins, both from Creality (Shenzhen, China). FDM process parameters were as follows: layer thickness 0.24 mm, nozzle diameter 0.4 mm, filament diameter 1.75 mm, printing temperature 215 °C, build platform temperature 65 °C, printing speed 60 mm/s. In the DLP process, the resin material was manufactured with the following parameters: layer height 0.05 mm, bottom layer count 10, exposure time 8 s, bottom exposure time 50 s, bottom lift distance 5 mm, bottom lift speed 65 s. DLP-printed specimens were only cleaned with alcohol and additionally cured, without polishing as a final process for removal of residual support.

### 2.2. Aging

The specimens were exposed to the natural environment and cleaned/washed daily for a duration of two months. This aging methodology was not used based on the standard, nor references, but with the intention to analyze the behavioral changes while “simulating” everyday indoor conditions (routine) in a rest state. Further research on the possible use of PLA resin as a material utilized for production of orthopedic immobilizators (i.e., for a toe) is planned. The aging conditions were as follows: specimens were stored in an open plastic box, with no exposure to direct sunlight, although they were on daylighting and artificial lighting during the night. The temperature was in the range of 17 to 25 °C due to the seasonal changes in the moment of the examination. Hygiene maintenance conditions included washing in 37 °C water using Frosch gel.

### 2.3. 3D Scanning

Scanning and geometrical accuracy checking of the thirty specimens took place immediately after manufacturing and two months later, using the Atos Core 200 (GOM, Braunschweig, Germany) non-contact 3D optical scanner. Fifteen specimens were made on the FDM device, and the other fifteen on the DLP one. Spraying was used for surface preparation of all thirty specimens to ensure better surface detection by the scanner. After scanning, ten specimens were tensile tested. Since the previous results showed insignificant changes in the PLA filament in the 7-day aging time [49], the current research focuses on scanning two groups of five specimens (FDM and DLP ones) right before mechanical testing, with the specimens having been manufactured two months before. The scanned STL models of specimens were compared in the 3D dimensional analysis software GOM Inspect 2020 software.

### 2.4. Mechanical Testing

Mechanical testing was performed for all 30 specimens on the Shimadzu AGS-X universal testing machine (Shimadzu Corp., Kyoto, Japan), equipped with a 100 kN load cell. The testing speed was set at 1 mm/min, following the ISO 527-2 standard. The average engineering stress–strain curves were computed in the Matlab R2022b software (MathWorks, Natick, MA, USA).

Research concerning the mechanical characterization of AM materials, especially in the field of tensile and compressive testing with different AM technologies and parameters, is important to broaden the knowledge and ensure better future designs [50].

### 2.5. Fourier Transform Infrared (FTIR) Spectroscopy

The Thermo Scientific Nicolet Summit FT-IR spectrometer with the ATR accessory and diamond crystal (Smart Orbit, Thermo Scientific, Madison, WI, USA) was used for IR spectra measurements. Spectral data were collected in the mid-IR range (4000–400 cm^−1^), with 32 scans and a 4 cm^−1^ resolution. A background spectrum was recorded with 32 scans. IR spectra were smoothed and baseline corrected, and an automatic ATR correction was performed using OMNIC software (version 7.0, Thermo Scientific, USA). Till now, the characterization of PLA materials was often carried out by FTIR spectroscopy, especially when natural or artificial aging processes were examined [44].

### 2.6. Statistical Analysis

The fact that material properties were observed in three time periods (immediate, 1 month, and 2 months) was the rationale for applying statistical analysis using Analysis of Variance (ANOVA), which is often used for data analysis of properties that are obtained from material tests [51,52,53]. Experimental results were statistically processed using the IBM SPSS 26 software package (IBM, Armonk, NY, USA).

The prerequisite for using the method is the homogeneity of the variance, which was tested using Levene statistics. While this condition was not met (Sig. < 0.05), the robust Welch and Brown–Forsythe tests, which are resistant to violations of assumptions, were used for these values.

## 3. Results and Discussions

### 3.1. Dimensional Analysis

To check the geometry and possible dimensional deviations between the modeled and printed specimens, as well as between the aged and non-aged specimens, 3D scans were carried out, and the data were compared. The first scan was immediately after printing. After two months, a 3D scan of the specimens was performed again, and this time, a comparison was made between the 3D scans of the same specimen scanned immediately after the production process and after the specified aging time.

**Figure 1:**
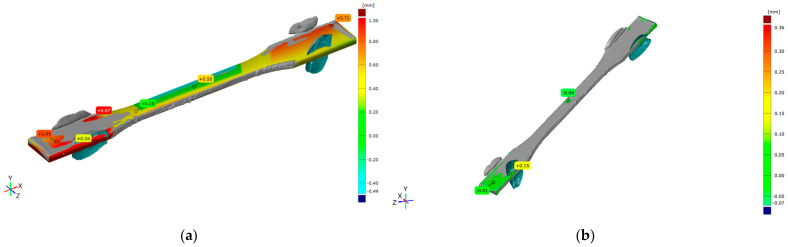
Comparison of 3D scan of tensile FDM non-aged specimen and (**a**) CAD model and (**b**) aged specimen.

**Figure 2:**
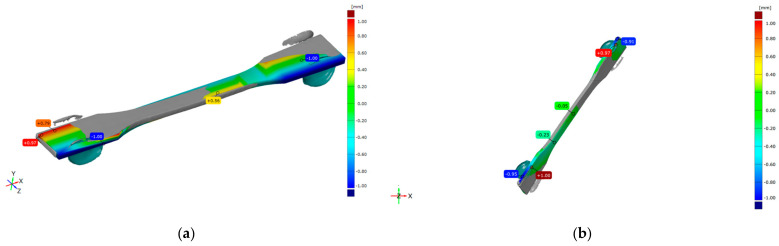
Comparison of 3D scan of tensile DLP non-aged specimen and (**a**) CAD model and (**b**) aged specimen.

Selected representations of overlapping CAD models and scanned specimens with marked deviations are shown in Figure 1 and Figure 2.

The deviations of the FDM tensile specimen scan from the corresponding CAD model are given in Figure 1a, and the measured deviations’ numerical range is [−0.49, +0.97] mm. The main reason for the deviation is the buckling of long and thin structures, such as the tensile specimen geometry.

A comparison of deviations between two 3D scans of the same FDM tensile specimens (non-aged and aged) is given in Figure 1b, showing the range deviation [−0.07, +0.36] mm. The highest value of +0.36 [-] is in a single spot, which is considered to be an error made by the used spray. Dominant green areas are not significant because of their negligible value of −0.04 [-]. The overall differences between non-aged and aged specimens’ geometry are insignificant.

The DLP tensile 3D scan’s deviations are in the range of [−1.0, +0.97] mm, as shown in Figure 2a, in contrast to the CAD model. A dimensional comparison between non-aged and aged 3D scans of the same specimen is given in Figure 2b, and the deviation range is [−1.0, +1.0] mm. In the case of on-edge printing, the orientation caused extensive deviation values here, as shown in Figure 2a, as well as in Figure 2b. The supports’ connectors at the edges caused red areas and material insufficiency, and at the opposite edges, they caused blue areas. Therefore, these positive extensive values could be eliminated from dimensional analyses. Dimensional analyses of the specimens before and after the aging process show a substantial change in the aged specimens.

From the previously mentioned data, it is obvious that the dimensional changes in the FDM specimen are negligible, while their geometry has remained unchanged. The dimensional analysis of the DLP specimens before and after the aging process shows that the dimensional accuracy is significantly affected in the case of long and thin specimens, such as the ones used for tensile testing.

### 3.2. FTIR Spectroscopy

**Figure 3:**
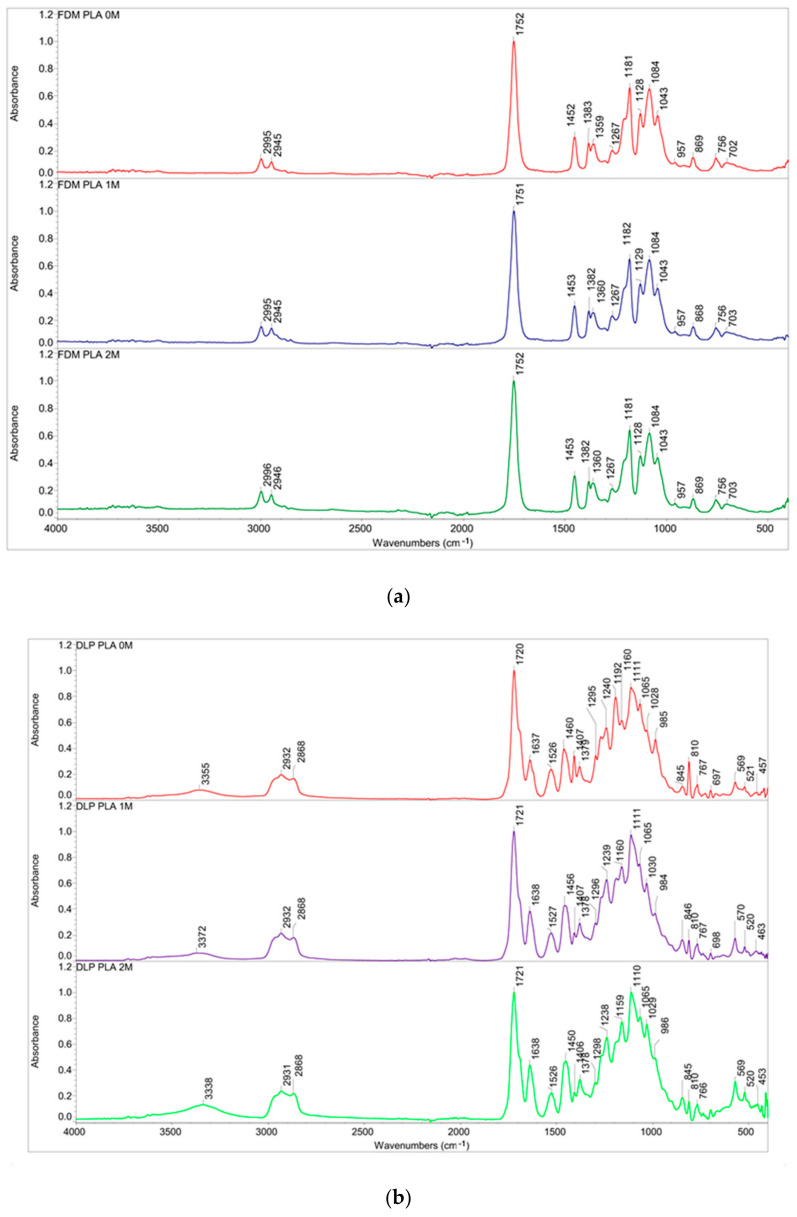
FTIR spectra comparison of three aged groups (i.e., immediately after specimen printing and after 1 and 2 months of aging) for (**a**) FDM and (**b**) DLP specimens.

The effect of the aging process on the polymer properties was monitored with FTIR spectroscopy on the FDM PLA filament and DLP PLA-like resin material. According to the presented results in the FTIR spectra of the DLP PLA-like material, known as polyurethane acrylate UV curing resin, N-H, C-H, and C=O stretching bands at 3342 cm^−1^, 2935 cm^−1^, and 1720 cm^−1^ were detected, respectively. Stretching C–O–C symmetric and asymmetric vibrations were observed in the ranges of 1050–1200 cm^−1^ and 1250–1300 cm^−1^ [54], and skeletal stretching at 1637 cm^−1^ from the CN Amide II was present. The peak at 1529 cm^−1^ shows in-plane NH deformation vibrations [55]. After printing, an alcohol rinsing procedure was mandatory. Even with this, the specimens exhibited sticky, rubbery-on-touch characteristics, meaning that the unpolymerized monomers of the resin remained on the surface. It was shown that residual monomers are present in resin-printed dentures, which significantly influenced the physical properties by affecting the surface characteristics, dimensional stability, water sorption, and compatibility [56]. The amount of residual monomer is inversely proportional to the degree of polymerization [57,58]. This can be seen in the FTIR spectra, where the intensity of the peaks changes noticeably right after printing and during the process of aging, i.e., washing with water. Regarding the FTIR spectrum of FDM PLA, the characteristic bands were as follows: CH_3_ vibrations from 2996 and 2944 cm^−1^, valention of C=O, and then C–O and C–O–C vibrations at 1747, 1266, and 1180 cm^−1^, respectively. The C–H deformation vibrations were detected at 1381 cm^−1^. The bands that were linked to an amorphous and crystalline phase were at 753 and 701 cm^−1^, respectively [59,60,61,62]. The aforementioned FTIR spectra are shown in Figure 3.

### 3.3. Statistical Analysis—Two-Way ANOVA

A two-way ANOVA was conducted to investigate the impact of the printing method and aging on the mechanical properties of PLA. Two printing methods were compared: FDM and DLP. The aging variable was defined at three predefined time points: non-aging (after specimen printing), after 1 month, and after 2 months. The following mechanical properties of PLA were observed: elastic modulus (MPa), ultimate tensile stress (MPa), elongation at yield (%), elongation at break (%), and toughness (J).

The analysis aimed to determine whether there are statistically significant main effects of the printing method, aging, and their interaction. Three null hypotheses were defined:

1. The main effect of printing method: There is no difference between the mean of observed mechanical properties when using FDM and DLP printing methods.
2. The main effect of the aging variable: There are no differences between the mean of observed mechanical properties when using FDM and DLP printing methods during aging (across the three time points).
3. Interaction effect: There is no interaction effect between printing method and aging on the observed mechanical properties when using FDM and DLP printing methods.

The mean values of the testing of the mechanical parameters obtained through two printing methods, FDM and DLP, during three time periods (immediately as “0m”, after 1 month as “1m”, and after 2 months as “2m”) are presented in Table 1.

Before applying the two-way ANOVA method, Levene’s Test of Equality of Error Variances was conducted, and since all values showed Sig. > 0.05, the condition for applying the method was met. In cases where this condition is not met (Sig. < 0.05), the robust Welch and Brown–Forsythe tests, which are resistant to violations of assumptions, were applied. Table 2 shows the two-way ANOVA test of between-subject effects for the maximum stress mechanical property.

The results of the two-way ANOVA test show that the elastic modulus, Max Stress, and Max Strain parameters are significantly influenced by the printing method, observation time, and their interaction (*p* < 0.0001 < 0.05). The Max Disp. Strain parameter is significantly influenced only by the Time variable (aging), Sig = 0.016 < 0.05. The impact of the printing method and the interaction of the printing method and Time is not statistically significant for this parameter, with *p*-values of 0.201 and 0.632, respectively, both exceeding the significance level of 0.05. The energy parameter is significantly influenced by the Time variable (aging) and printing time (with *p*-values < 0.05, of 0.001 and 0.000, respectively), but not by their interaction.

**Figure 4:**
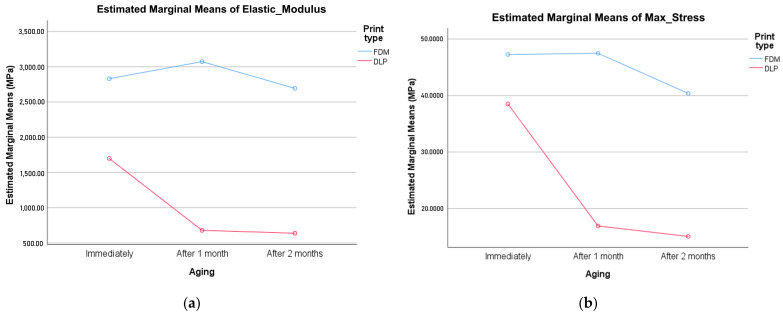

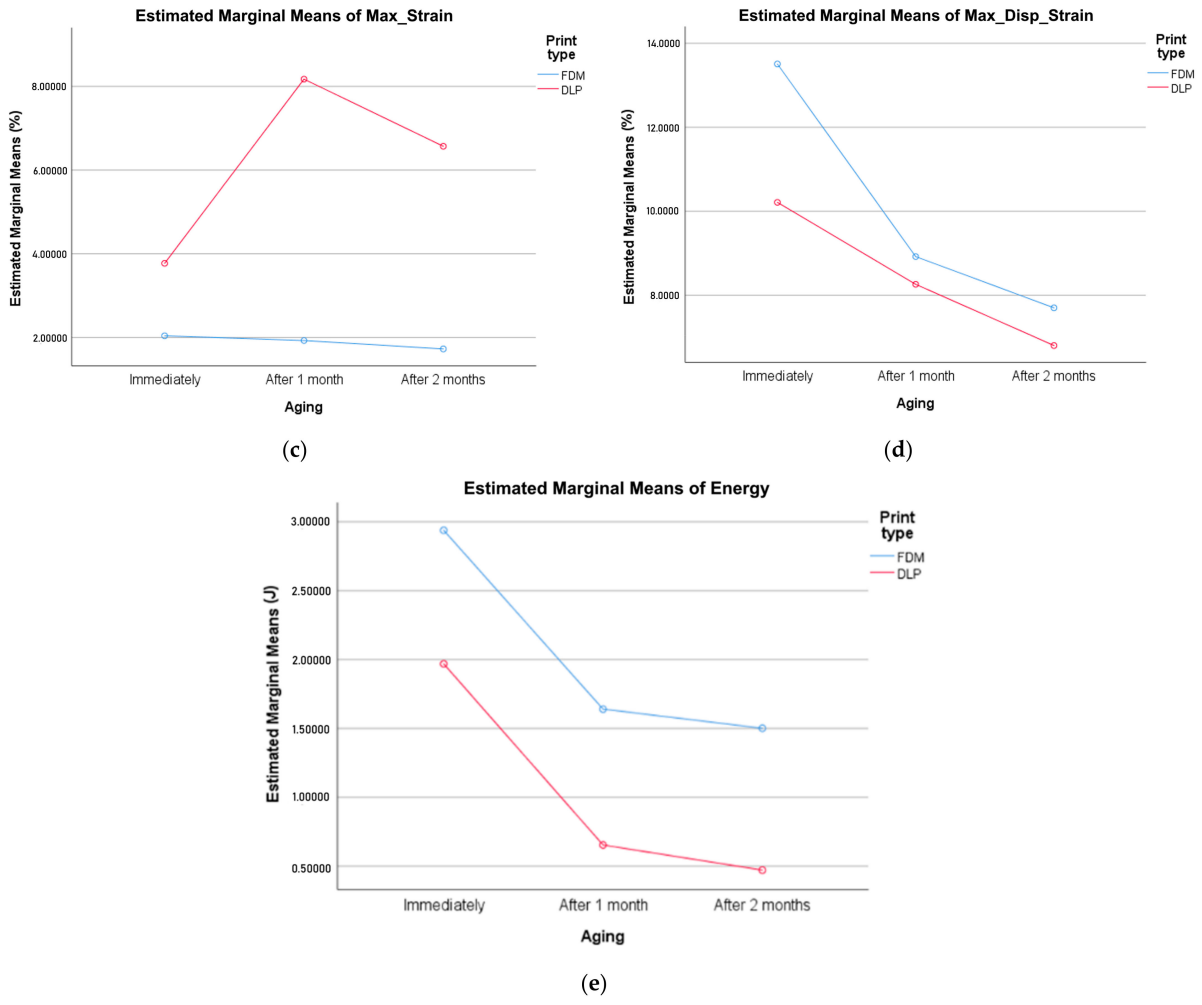
FDM and DLP specimens’ aging: (**a**) elastic modulus; (**b**) ultimate tensile stress; (**c**) elongation at yield (%); (**d**) elongation at break; (**e**) toughness.

Reviewing the line graphs of the group means is the easiest way to get an overview of the results of a two-way ANOVA and to assess whether there is an interaction between independent variables. When the lines in the plots are clearly not parallel to each other, this indicates that there is a significant interaction effect. When the lines in the profile group are approximately parallel to each other, this indicates that there is no significant interaction effect. Figure 4a,b illustrates the nature of these differences, showing that higher values for elastic modulus and Max Stress were obtained for the FDM printing method. Figure 4c shows that higher values for Max Strain were obtained for the DLP printing method. Figure 4d shows that the MaxDispStrain for both printing methods, FDM and DLP, decreases over time. In Figure 4e, it is evident that both graphs are nearly parallel, and the energy parameter decreases over time. However, higher values are obtained for the FDM printing method.

### 3.4. Mechanical Property Comparison

**Figure 5:**
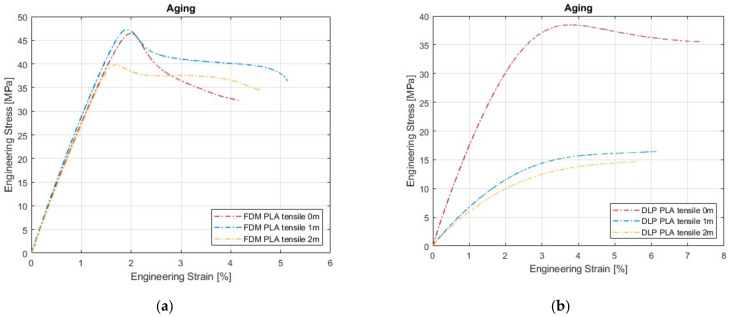
Tensile curve comparison of aging for (**a**) FDM and (**b**) DLP specimens.

Tensile testing was performed on 30 specimens, arranged in groups of 5 per testing session. Exactly half of the specimens were produced by FDM printing, and the other half was DLP PLA specimens. Further, each half was then partitioned according to aging: immediately after the AM process (denoted as “0m”), after one month of aging (denoted as “1m”), and after two months of aging (denoted as “2m”). The raw data from the tensile testing machine were then processed using Matlab software. Figure 5 presents the average stress–strain value curves for all five specimens in the group. The average curves for FDM specimens are presented in Figure 5a, and for DLP specimens, they are presented in Figure 5b. It is worth noting that the averaging procedure was based on the simple mean value of the curves, and the interpolation of the data was not considered. Hence, the average curves were created based on the interval where all five specimens had available data. The interval after the first specimen failure and onward was not considered. The idea here was just to point out the trend in the stress–strain values of all six data groups.

PLA is not water-soluble, and filaments do not show any reaction to water when it is applied on specimens after printing. However, in the case of PLA resin, when specimens are printed, as mentioned before, the surface of the specimens is sticky. When washed with water, the stickiness is removed, which means that non-polymerized monomers from the specimens’ surface are washed away, since debris was observed in the water during the initial washing process.

The average curves for the FDM material (Figure 5a) reveal an unexpected behavior for the month-old group, since it performs a bit better than the brand-new material.

The maturation of the thermoplastic polymer occurred in warm water bathing during a period of one month, which might have influenced the outcome. The group that aged for two months exhibited a worse mechanical response, which was anticipated.

Figure 5b shows a clear difference in the stress–strain trend between the brand-new material and the aged ones. The worse mechanical response from the aged specimens was anticipated.

For FDM specimens, aging and bathing led to fluctuations in the following properties:

(1) Ultimate tensile stress—fluctuating less than 15%,
(2) Elongation at yield—around 15%,
(3) Elastic modulus—around 12% (acc. to Table 1).

For DLP specimens, the following was noticed:

(1) Ultimate tensile stress decreased by 61%,
(2) Elongation at yield fluctuated around 54%,
(3) Elastic modulus decreased by 62%.

Observing the values from Table 1, one can see that aging and warm water bathing led to significant changes in the tensile properties of the PLA material, manufactured by DLP. The decrease in mechanical response is evident in the two-month-aged FDM specimens.

## 4. Conclusions

AM is a fast-developing technology, continuously pushing the boundaries of modern manufacturing capabilities. However, the data on the effect of aging on AM materials are still scarce, especially with newly introduced materials, such as PLA-like resin. This research aims to determine the differences in the mechanical, chemical, and geometrical properties of two forms of PLA material, all with an aging period of two months. The applied approach regarding the comparison of PLA filament and resin polymer material adds to the existing literature by employing mechanical testing, 3D scanning, Fourier transform infrared spectroscopy (FTIR), and two-way ANOVA statistical analysis. Ambient aging and watering were performed to predict the possible in vitro behavior of both PLA material types, with a special emphasis on the resin counterpart, which is a candidate material for future orthopedic immobilizations.

The dimensional analysis of brand-new and aged tensile specimens shows significant impairment in the dimensional accuracy of long and thin parts. Taking into account the tensile results from this study, the FDM parts are less prone to aging degradation than the DLP ones. The aforementioned conclusion relies on the insignificant difference in the mechanical response between the brand-new and one-month-aged FDM groups. Based on the analyses performed, it can be concluded that the time interval of two months, during which the specimens were exposed to water, led to a change in the physical and mechanical properties of the DLP specimens in particular. The FTIR spectra confirm the influence of aging, structural changes, and degradation of the PLA material, especially in the case of the resin-based specimens. The results of the two-way ANOVA test show that aging has a significant influence on the elastic modulus, ultimate tensile stress, elongation at yield, elongation at break, and toughness.

This research will contribute to recognizing the mechanical and chemical changes that are induced by aging based on various analytical and characterization techniques. Hence, the data collected will lead to a better understanding of the underlying aging mechanism of PLA materials.

## Figures and Tables

**Table 1:** Mechanical parameters for tensile testing, descriptive statistics.

Printing Method	Aging	N	Elastic Modulus (MPa)		Max_Stress (MPa)		Max Strain (%)		MaxDispStrain (%)		Energy (J)	
			Mean	Std.Dev	Mean	Std.Dev	Mean	Std.Dev	Mean	Std.Dev	Mean	Std.Dev
**FDM**	0m	5	2830.29	136.379	47.2603	0.42555	2.03999	0.12780	13.5074	5.77845	2.93832	1.41310
	1m	5	3071.68	46.6987	47.4790	3.36573	1.92660	0.10414	8.92187	3.77949	1.63997	0.86457
	2m	5	2693.18	36.0283	40.3750	2.12914	1.72720	0.14180	7.70198	2.24038	1.50123	0.52255
	Total	15	2865.05	180.382	45.0381	4.02997	1.89793	0.17726	10.0437	4.66350	2.02650	1.14496
**DLP**	0m	5	1698.15	84.4700	38.5189	1.78268	3.77121	0.08383	10.2137	3.21002	1.96871	0.61411
	1m	5	678.61	118.174	16.9080	1.66109	8.17447	1.79453	8.26229	1.81334	0.65386	0.11958
	2m	5	638.48	104.179	15.0601	2.25315	6.57026	1.44566	6.80498	1.34847	0.47149	0.18461
	Total	15	1005.08	516.474	23.4956	11.1653	6.17198	2.25088	8.42700	2.54805	1.03135	0.77344
**Total**	0m	10	2264.22	606.197	42.8896	4.76639	2.90560	0.91810	11.8605	4.73638	2.45352	1.14728
	1m	10	1875.14	1264.10	32.1935	16.3054	5.05054	3.50419	8.59208	2.81620	1.14691	0.78018
	2m	10	1665.83	1085.41	27.7175	13.5012	4.14873	2.73004	7.25348	1.80623	0.98636	0.65654
	Total	30	1935.06	1019.40	34.2669	13.7129	4.03496	2.68056	9.23538	3.78279	1.52893	1.08525

**Table 2:** Tensile test, ANOVA tests of between-subject effects, dependent variable: Max_Stress (N).

Source	Type III Sum of Squares	df	Mean Square	F	Sig.	Partial Eta Squared
Corrected Model	53,45.046 ^a^	5	1069.009	237.062	0.000	0.980
Intercept	35,226.613	1	35,226.613	7811.811	0.000	0.997
Print_type	3480.580	1	3480.580	771.849	0.000	0.970
Time	1215.434	2	607.717	134.767	0.000	0.918
Print_type * Time	649.033	2	324.517	71.964	0.000	0.857
Error	108.226	24	4.509			
Total	40,679.885	30				
Corrected Total	5453.272	29				

^a^ R Squared = 0.980 (Adjusted R Squared = 0.976).

## Data Availability

Data can be requested from via the corresponding author.
